# No significant benefit of moderate-dose vitamin C on severe COVID-19 cases

**DOI:** 10.1515/med-2021-0361

**Published:** 2021-09-22

**Authors:** Shaoping Zheng, Qiaosen Chen, Hongbo Jiang, Chunxia Guo, Jinzhuo Luo, Sumeng Li, Hua Wang, Huadong Li, Xin Zheng, Zhihong Weng

**Affiliations:** Department of Ultrasound, Union Hospital, Tongji Medical College, Huazhong University of Science and Technology, Wuhan, China; Department of Epidemiology and Biostatistics, School of Public Health, Guangdong Pharmaceutical University, Guangzhou, China; Department of Infectious Diseases, Union Hospital, Tongji Medical College, Huazhong University of Science and Technology, Wuhan, China; Department of Infectious Diseases, Wuhan Jinyintan Hospital, Wuhan, China; Joint International Laboratory of Infection and Immunity, Huazhong University of Science and Technology, Wuhan, China

**Keywords:** SARS-Cov-2, COVID-19, vitamin C

## Abstract

There is no specific drug for coronavirus disease 2019 (COVID-19). We aimed to investigate the possible clinical efficacy of moderate-dose vitamin C infusion among inpatients with severe COVID-19. Data of 397 adult patients with severe COVID-19 admitted to a designated clinical center of Wuhan Union Hospital (China) between February 13 and February 29, 2020, were collected. Besides standard therapies, patients were treated with vitamin C (2–4 g/day) or not. The primary outcome was all-cause death. Secondary outcome was clinical improvement of 2 points on a 6-point ordinal scale. About 70 participants were treated with intravenous vitamin C, and 327 did not receive it. No significant association was found between vitamin C use and death on inverse probability treatment weighting (IPTW) analysis (weighted hazard ratio [HR], 2.69; 95% confidence interval [CI], 0.91–7.89). Clinical improvement occurred in 74.3% (52/70) of patients in the vitamin C group and 95.1% (311/327) in the no vitamin C group. No significant difference was observed between the two groups on IPTW analysis (weighted HR, 0.76; 95% CI, 0.55–1.07). Our findings revealed that in patients with severe COVID-19, treatment with moderate dose of intravenous vitamin C had no significant benefit on reducing the risk of death and obtaining clinical improvement.

## 1 Introduction

The coronavirus disease 2019 (COVID-19) caused by severe acute respiratory syndrome coronavirus 2 (SARS-CoV-2) has led to a pandemic around the world since first reported in Wuhan, China, in December 2019 [1]. COVID-19 has considerable high morbidity and mortality and has caused severe public health burdens. To date, there is a lack of specific drugs for COVID-19. An effective and safe treatment is urgently needed to save patients with COVID-19 and curtail the pandemic.

Similar to influenza viruses, coronavirus infection usually results in increased oxidative stress leading to the production of free radicals and cytokines, which is the characteristic of acute respiratory distress syndrome (ARDS) [2], the main mechanism of COVID-19’s fatality [1]. Vitamin C is regarded as an antioxidant and is thought to enhance immune function [3]. It is a safe and inexpensive essential nutrient. A relatively low dose of supplemental vitamin C may save lives. Previously, a randomized study showed that 200 mg of vitamin C per day when given to severely ill respiratory disease inpatients resulted in an 80% decrease in deaths [4]. It has been demonstrated that vitamin C levels decreased obviously in critically ill patients (e.g., septic shock) despite receiving enteral and parenteral nutritional support [5]. The intake of vitamin C (1–4 g/day) can increase the levels of vitamin C to the normal range in patients with multiple organ dysfunction [6]. Moreover, a meta-analysis of 1,766 patients in ICU found that vitamin C can shorten ICU stay by 7.8% [7]. Another meta-analysis of eight trials concluded that vitamin C can reduce the length of mechanical ventilation by 14% in critically ill patients [8]. Therefore, we hypothesized that vitamin C could serve as a potential therapy for COVID-19 patients [9].

However, clinical data on the effect of vitamin C use in patients with COVID-19 are scarce. This study aimed to investigate the clinical efficacy of moderate-dose vitamin C infusion by using the Cox regression analysis with inverse probability of treatment weighting (IPTW) and propensity-score matching among inpatients with severe COVID-19.

## 2 Methods

### 2.1 Study design

Data of all patients with COVID-19 consecutively admitted to the Cancer Centre, a designated clinical center for COVID-19 of the Union Hospital of Huazhong University of Science and Technology (Wuhan, China) between February 13 and February 29, 2020, were collected. Patients aged ≥18 years and diagnosed as severe COVID-19 were screened for this study. Exclusion criteria were (a) the duration of hospitalization was less than 3 days; (b) vitamin C treatment started before admission; and (c) the length of vitamin C use was less than 3 days. Definite outcomes (discharge or death) were followed up until March 15, 2020.

All patients in this study were diagnosed according to the Guidelines of the Diagnosis and Treatment of Novel Coronavirus Pneumonia released by the China NHC [10]. The definition of severe COVID-19 was patients who met any of the following criteria: (a) demonstrating shortness of breath with a respiratory rate ≥30 beats/min; (b) pulse oxygen saturation (SpO_2_) ≤93% in resting state; (c) arterial blood oxygen partial pressure (PaO_2_)/oxygen concentration (FiO_2_) ≤300 mm Hg; (d) pulmonary imaging demonstrating significant >50% increase of lesions within 24–48 h.

Data on demographic characteristics, clinical features, comorbidities, laboratory findings, medications, and outcomes were obtained from the patients’ electronic medical records and evaluated by two trained physicians (CXG and JZL) independently. This study was approved by the Ethics Committee of Tongji Medical College of Huazhong University of Science and Technology in Wuhan (2020-0058). Written informed consent was waived due to the rapid emergence of this infectious disease.

### 2.2 Vitamin C exposure

Patients treated with intravenous vitamin C (2–4 g/day) at study baseline or received it during follow-up before death or discharge were defined as vitamin C exposure. The study baseline for each patient was 24 h after admission. As there was no powerful evidence for vitamin C use in patients with COVID-19, the duration of vitamin C treatment was not standardized and was determined by the clinicians according to the condition of each patient.

### 2.3 Outcomes

The primary outcome was all-cause death during the follow-up period. The secondary outcome was clinical improvement assessed at the end of follow-up. Clinical improvement was defined as live discharge from the hospital, or a reduction of at least 2 points from baseline on a 6-point ordinal scale [11], whichever occurred first.

The 6-point ordinal scale was as follow: (1) discharge; (2) hospitalized, breathing ambient air without supplemental oxygen; (3) hospitalized, receiving low-flow supplemental oxygen; (4) hospitalized, requiring noninvasive mechanical ventilation or high-flow oxygen; (5) hospitalized, requiring invasive ventilation or extracorporeal membrane oxygenation (ECMO); and (6) death [11].

### 2.4 Statistical analysis

Discrete variables were presented as frequency and proportion, and the continuous were described using median and interquartile range (IQR). First, we applied univariate Cox proportional-hazards regression models to evaluate the association between vitamin C use and outcomes. For controlling the potential confounders, two propensity-score based strategies were adopted, one of which is IPTW and another is 1:1 propensity-score matching. The propensity score concerning the assignment of vitamin C was modelled by a non-parsimonious multivariable logistic regression incorporating patients’ demographics, comorbidities, laboratory testing, and medications at study baseline. We assessed the distribution of the weights as well as the propensity scores and then conducted the covariate balance diagnostics using standardized differences with the threshold of 0.25 [12]. In IPTW analysis, we adjusted the weights of samples by basic stabilized inverse probability treatment weights without any truncation. The weighted hazard ratios (HR) were estimated using the Cox model, and Kaplan–Meier curves were generated to compare survival rates of the patients in different groups. In 1:1 matching analysis, we matched the patients using the nearest neighbor matching with the caliper of 0.2, after which we clustered every single pair in the Cox model to improve the statistical power.

We tested the proportional assumption of the Cox model based on Schoenfeld residuals against the transformed time and found no evidence of violation during the analysis. For paired survival analysis, the robust variance was estimated. Multiple imputations were used for the missing data at the study baseline, and the coefficients and standard errors were all estimated based on Rubin’s rules. All statistical analyses were performed with the software R version 4.0.2 (https://www.r-project.org/).

## 3 Results

### 3.1 Characteristics of the patients

**Figure 1:**
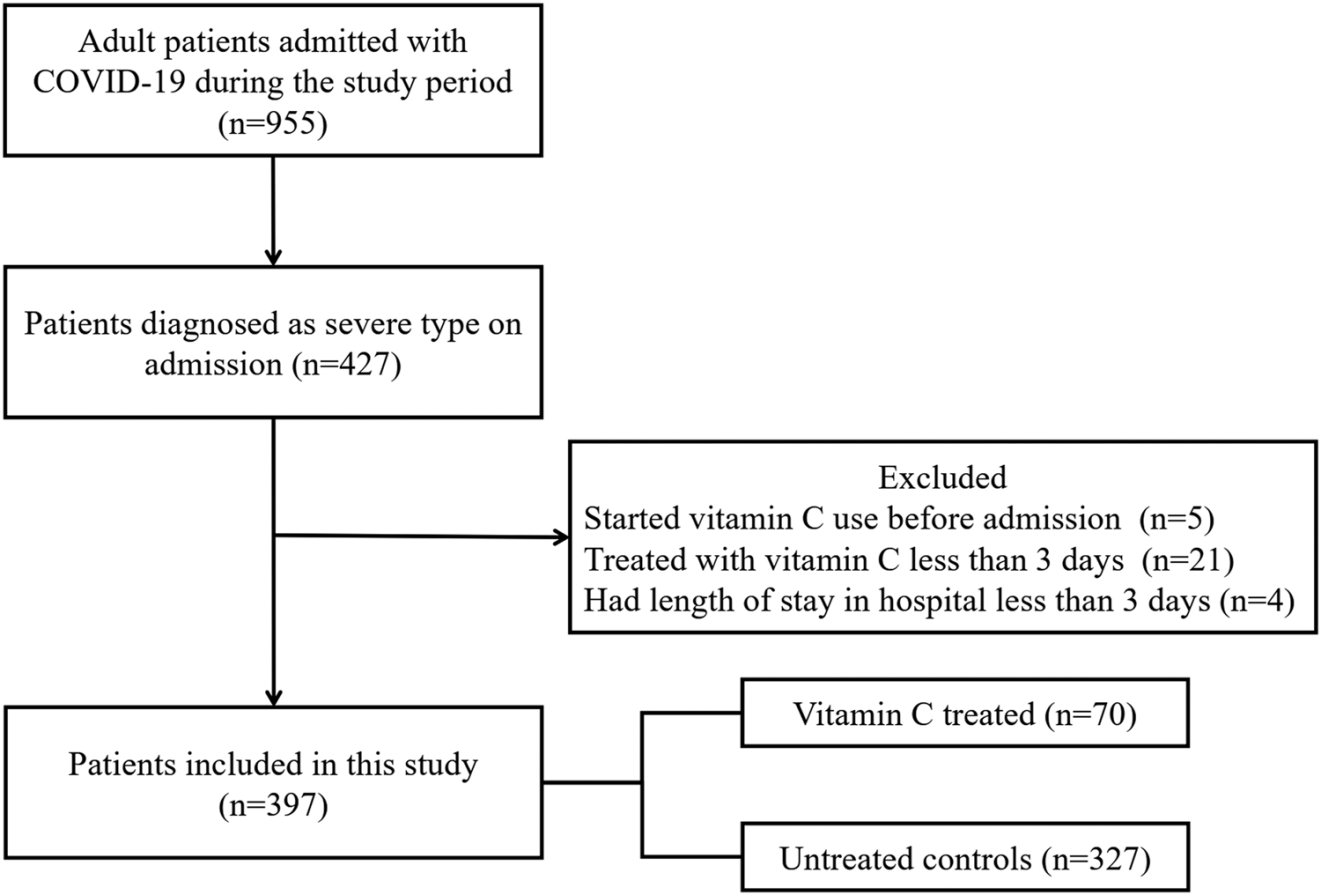
Flow chart of study participants in the study cohort. The study baseline was defined as 24 h after admission. COVID-19 denotes coronavirus disease 2019.

Among 955 patients with COVID-19 who were admitted consecutively to the hospital between February 13 and February 29, 2020, 427 were diagnosed as severe type on admission. Of the 427 patients, among which 30 were excluded from the study (5 started vitamin C use before admission, 21 were treated with vitamin C for less than 3 days, and 4 had a length of stay in hospital of less than 3 days) (Figure 1).

**Figure A1:**
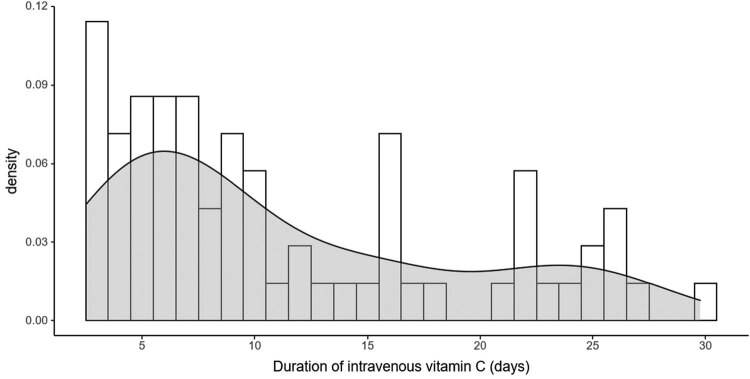
Distribution of duration of vitamin C use for the patients who received it.

**Figure A2:**
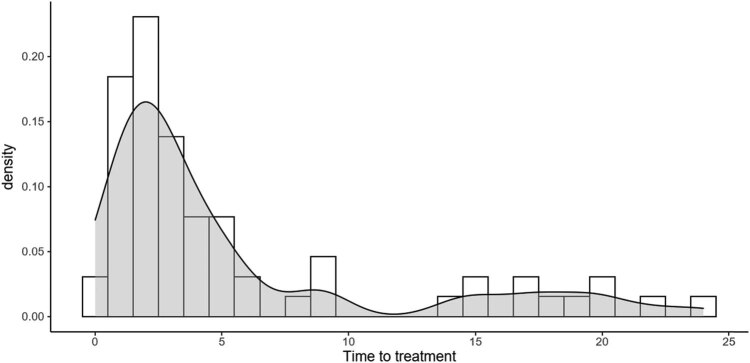
Distribution of time (in days) from hospital admission to the initiation of vitamin C use for the patients who received it.

Of the 397 patients, in addition to standard therapies, 70 (17.6%) received intravenous vitamin C (2–4 g/day) and 327 (82.4%) did not. The median duration of vitamin C treatment was 8.8 days (interquartile range [IQR], 5.1–16.0 days). The distribution of duration of vitamin C use is shown in Figure A1. Among the 70 patients who were exposed to vitamin C, 33 (47.1%) received it within 48 h of admission. The timing of the initiation of vitamin C use after admission is provided in Figure A2. The baseline demographic and clinical characteristics of the patients with severe COVID-19 according to vitamin C exposure are shown in Table 1.

**Table 1:** Baseline characteristics of patients who received vitamin C or not, before and after inverse probability treatment weighting (IPTW)

Characteristics	Unweighted patients				Weighted patients			
	Yes	No	Overall	Absolute SMD	Yes	No	Overall	Absolute SMD
	(*N* = 70)	(*N* = 327)	(*N* = 397)		(*N* = 73)	(*N* = 323)	(*N* = 396)	
Age, median (IQR), *y*	67.50 (58.00–74.75)	67.00 (62.00–74.00)	67.00 (61.00–74.00)	0.08	69.97 (64.97–75.90)	67.00 (62.00–74.00)	68.00 (62.00–74.00)	0.10
Sex, No. (%)								
Female	28 (40.0)	162 (49.5)	190 (47.9)	0.19	34.9 (47.9)	153.8 (47.7)	188.8 (47.7)	0.01
Male	42 (60.0)	165 (50.5)	207 (52.1)		38.0 (52.1)	168.8 (52.3)	206.8 (52.3)	
Comorbidity, No. (%)								
Hypertension	13 (18.6)	70 (21.4)	83 (20.9)	0.07	22.7 (31.1)	69.5 (21.5)	92.2 (23.3)	0.24
Coronary heart disease	3 (4.3)	22 (6.7)	25 (6.3)	0.10	3.8 (5.3)	19.6 (6.1)	23.5 (5.9)	0.04
Chronic lung disease^a^	4 (5.7)	15 (4.6)	19 (4.8)	0.05	2.8 (3.8)	15.7 (4.9)	18.5 (4.7)	0.05
Cerebral infarction	2 (2.9)	8 (2.4)	10 (2.5)	0.03	3.8 (5.2)	9.2 (2.8)	12.9 (3.3)	0.15
Diabetes	11 (15.7)	51 (15.6)	62 (15.6)	<0.01	12.1 (16.6)	50.0 (15.5)	62.1 (15.7)	0.03
Cancer	6 (8.6)	16 (4.9)	22 (5.5)	0.16	3.3 (4.6)	17.3 (5.4)	20.6 (5.2)	0.04
Initial laboratory tests, median (IQR)^b^								
Neutrophil (×10^9^/L)	4.70 (3.29–6.79)	3.46 (2.72–4.69)	3.62 (2.72–5.03)	0.66	3.94 (2.87–5.12)	3.57 (2.73–4.92)	3.67 (2.73–5.00)	0.05
Lymphocyte (×10^9^/L)	0.92 (0.62–1.33)	1.23 (0.84–1.59)	1.14 (0.81–1.58)	0.32	1.08 (0.86–1.79)	1.18 (0.81–1.58)	1.17 (0.81–1.60)	0.02
Lactate dehydrogenase (U/L)	276.00 (213.00–370.00)	212.00 (175.25–264.75)	219.00 (181.00–280.50)	0.76	225.35 (198.06–280.00)	216.59 (179.00–273.88)	219.00 (184.00–276.28)	0.09
C-Reactive protein (mg/L)	25.55 (7.13–99.10)	7.19 (3.14–33.58)	10.07 (3.14–39.67)	0.65	14.00 (3.14–30.83)	8.99 (3.14–41.19)	10.80 (3.14–38.99)	0.02
Alanine aminotransferase (U/L)	27.00 (17.00–41.00)	25.00 (17.00–39.00)	25.00 (17.00–40.00)	0.06	23.29 (16.00–50.52)	26.00 (18.00–41.00)	26.00 (17.00–42.17)	0.06
Creatinine (μmol/L)	74.00 (61.40–99.20)	74.00 (63.90–86.17)	74.00 (63.00–88.00)	0.14	77.09 (62.71–90.40)	73.46 (63.00–86.00)	74.00 (63.00–86.70)	0.03
D-dimer (mg/L)	1.17 (0.33–4.22)	0.75 (0.32–1.84)	0.82 (0.32–2.04)	0.40	1.12 (0.20–1.90)	0.82 (0.34–2.01)	0.84 (0.32–1.92)	0.05
Medications at baseline, no. (%)								
Arbidol	62 (88.6)	300 (91.7)	362 (91.2)	0.11	66.8 (91.5)	295.7 (91.7)	362.5 (91.6)	<0.01
Interferon α-2b	25 (35.7)	34 (10.4)	59 (14.9)	0.71	12.6 (17.3)	46.7 (14.5)	59.3 (15.0)	0.08
Ribavirin	21 (30.0)	37 (11.3)	58 (14.6)	0.53	11.0 (15.1)	46.9 (14.5)	57.8 (14.6)	0.02
LMWH	23 (32.9)	53 (16.2)	76 (19.1)	0.42	16.5 (22.7)	58.8 (18.2)	75.3 (19.0)	0.11
Antibiotics	64 (91.4)	248 (75.8)	312 (78.6)	0.38	56.3 (77.3)	252.8 (78.3)	309.1 (78.1)	0.03
ARB/ACEI	5 (7.1)	37 (11.3)	42 (10.6)	0.14	6.1 (8.4)	33.8 (10.5)	39.9 (10.1)	0.07
Chinese medicine	56 (80.0)	271 (82.9)	327 (82.4)	0.08	57.1 (78.3)	266.0 (82.5)	323.2 (81.7)	0.11

Abbreviations: SMD, standardized mean difference; IQR, interquartile range; LMWH, low molecular weight heparin; ARB, angiotensin-receptor blocker; ACEI, angiotensin-converting enzyme inhibitor.

^a^Chronic lung disease was defined as a chronic obstructive pulmonary disease (COPD), asthma, or chronic bronchitis.

^b^In the unweighted analysis, data on the D-dimer level were missing for 65 patients, on the C-reactive protein level for 11, on the lactate dehydrogenase level for 6, on the creatinine level for 4, on the ALT for 4, on the neutrophil count for 3, and on the lymphocyte count for 3. Multiple imputation was used to handle missing data in the inverse probability treatment weighting analysis.

In the unadjusted population, the median age of the patients was 67.0 (IQR, 61.0–74.0) years, and 207 (52.1%) were men. Vitamin C-treated patients had a higher level of C-reactive protein at study baseline than the patients who did not receive it. Interferon α-2b was administered to 35.7% of the patients in the vitamin C treatment group versus 10.4% in the control group, and low molecular weight heparin was given to 32.9% versus 16.2%, respectively (Table 1).

**Figure A3:**
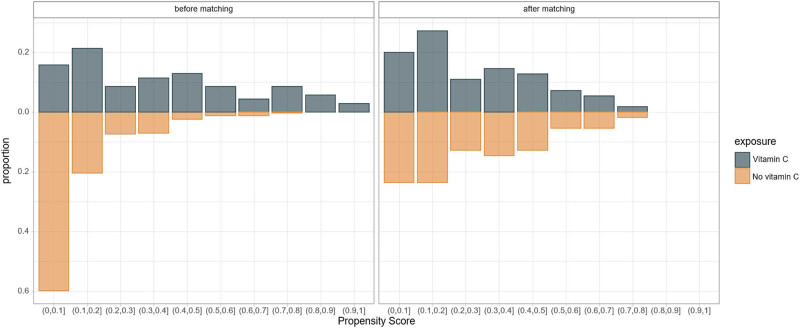
Distribution of the estimated propensity score for receiving vitamin C among patients enrolled in the study.

**Figure A4:**
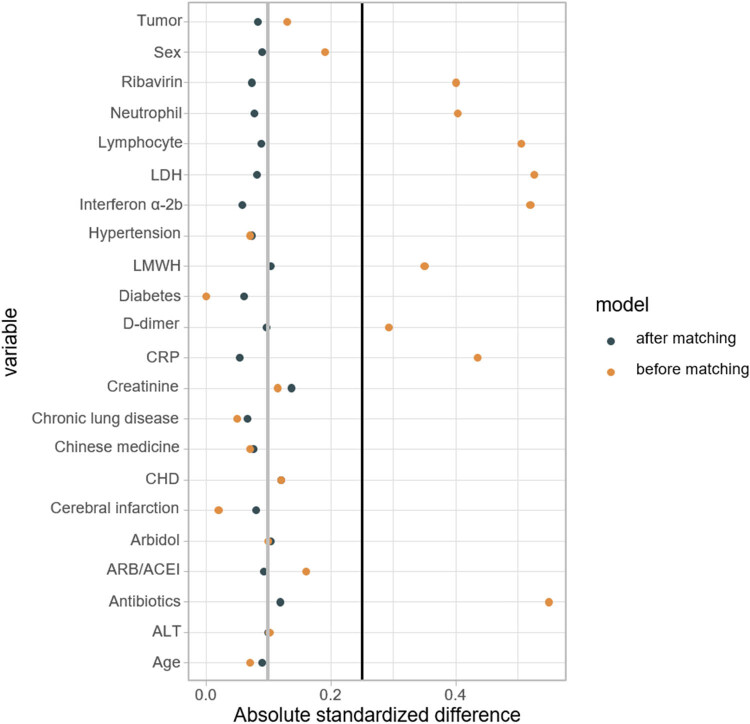
Standardized mean differences of variables in the propensity-score matching analysis.

**Figure A5:**
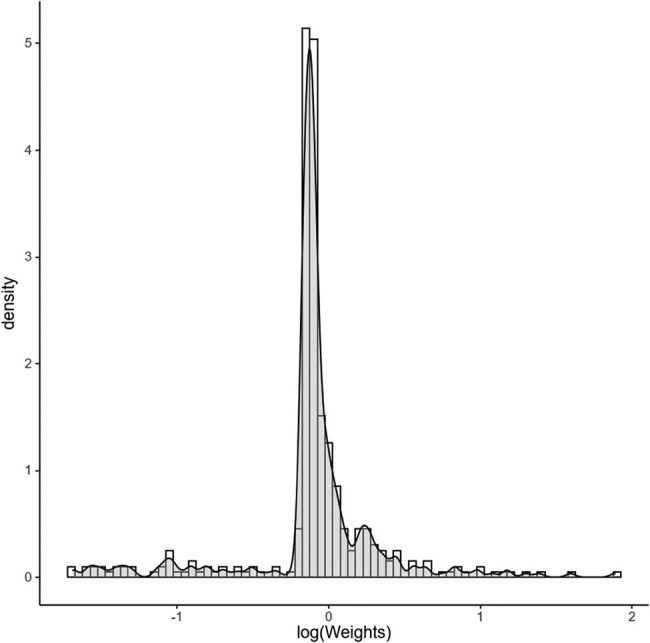
Distribution of weights in inverse probability treatment weighting analysis.

**Figure A6:**
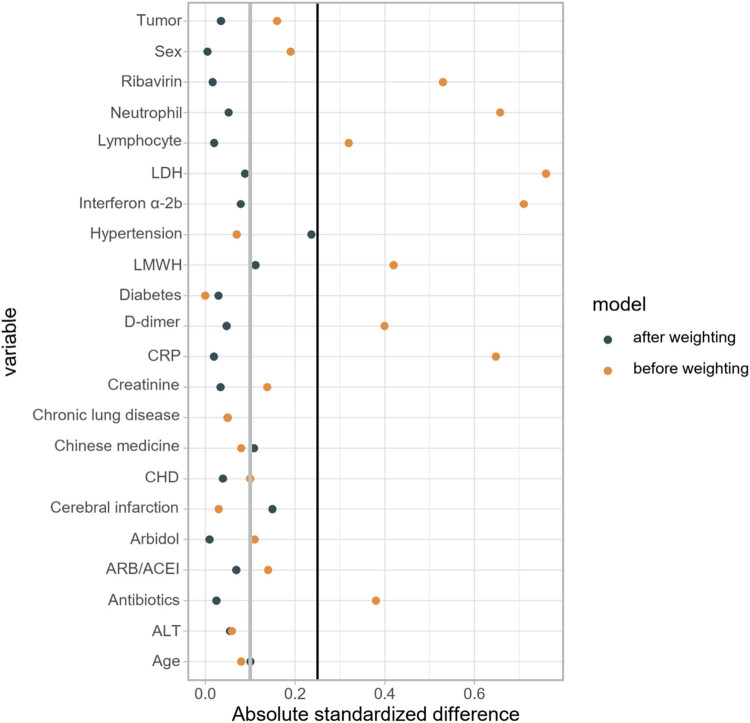
Standardized mean differences of variables in inverse probability treatment weighting analysis.

Figure A3 shows the distribution of estimated propensity scores for the assignment of intravenous vitamin C among the severe COVID-19 population before and after matching. After matching, there is a balanced propensity score distribution between the exposed and unexposed groups. Particularly, the absolute standardized mean difference (aSMD) of the variables incorporated in the propensity score model indicated that it is of comparability between two groups (aSMD <0.25) (Figure A4). Figure A5 displays the distribution of weights for IPTW analysis on a log scale. Particularly, the mean of stabilized weighting is approximately 1, indicating that we did not observe the obvious weighting discrepancy from the theoretical framework after standardization. Also, the maximum of the weighting is less than 10 and the minimum of the weighting is greater than 0.1, which shows us less risk of positivity assumption violation. Hence, we did not observe the distinct evidence indicating the absence of the IPTW assumption violation. Figure A6 also reflects that the two groups are comparable based on the threshold of 0.25 for aSMD.

### 3.2 Primary outcome

Over a median follow-up of 29.3 days (IQR, 28.5–30.1 days), the primary outcome developed in 19 patients (4.8%) of the 397 enrolled in our study. At the end of follow-up on 15 March 2020, 360 (90.7%) patients were discharged alive, and 18 (4.5%) were still in the hospital.

**Figure 2:**
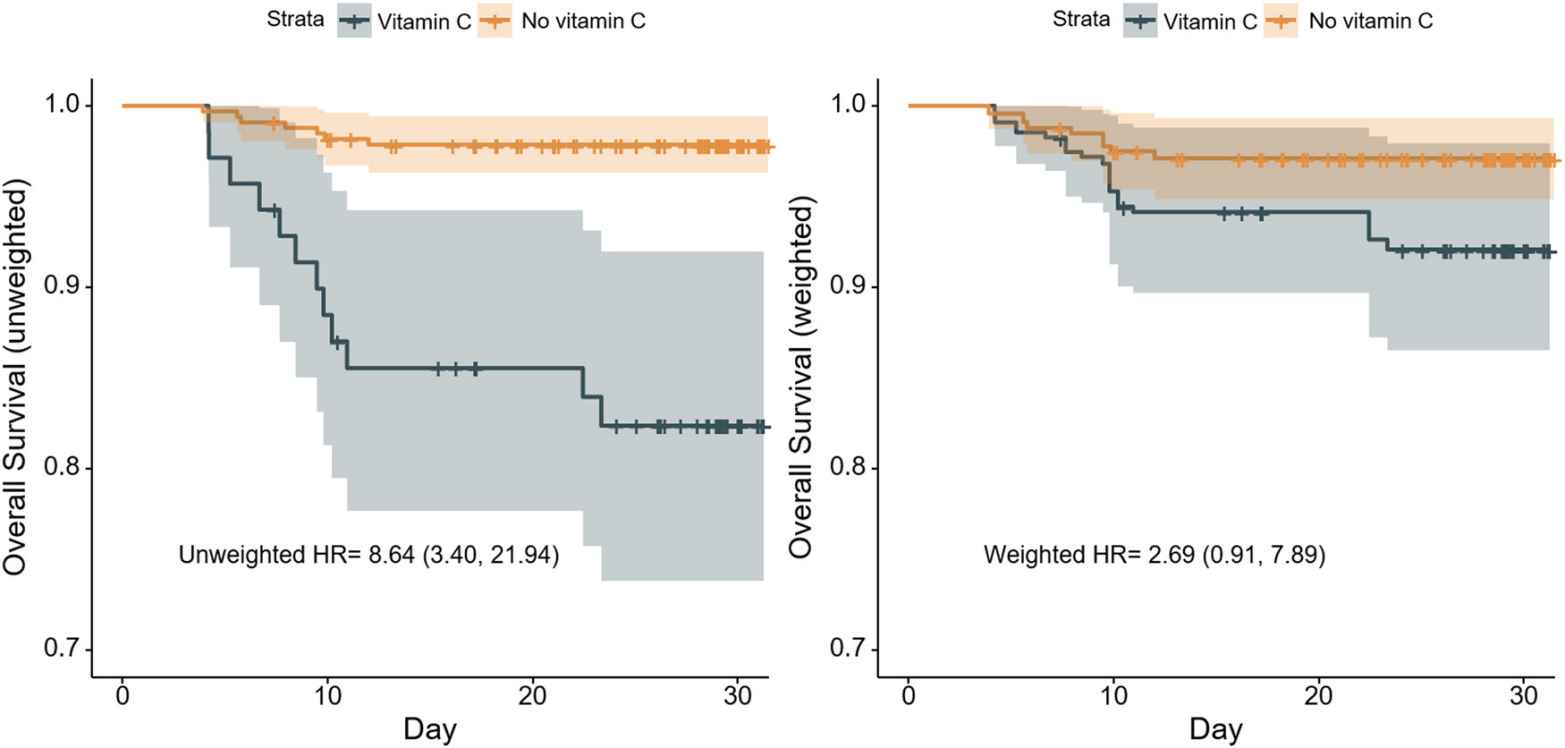
Kaplan–Meier curves for overall survival during follow-up before and after inverse probability of treatment weighting (IPTW) analysis. On unadjusted analysis in the left panel, patients treated with vitamin C had higher risk of death than those who did not receive it (HR, 8.64; 95% CI, 3.40–21.94), while there was no significant association between vitamin C use and death on IPTW analysis in the right panel (weighted HR, 2.69; 95% CI, 0.91–7.89). The shaded areas represent 95% CI.

Among 397 patients in two groups, in contrast to 0.206 deaths/person-months in the population receiving intravenous vitamin C, those not receiving vitamin C is 0.02 deaths/person-months only. In the crude analysis, according to the Kaplan–Meier curves in the left panel of Figure 2, it seems to be a significant difference between the two groups (HR, 8.64; 95% confidence interval [CI], 3.40–21.94). However, after adjusting potential confounders, IPTW analysis (Figure 2, right panel) shows the weighted HR and its 95% CI as 2.69 (0.91–7.89). A similar non-significant result could also be found in the 1:1 matching analysis. After matching, the paired Cox model indicates that the HR is 2.57 (95% CI, 0.39–16.79) (Table 2), without significance.

**Table 2:** Associations between vitamin C use and death or clinical improvement in the crude analysis and propensity-score analyses

Analysis	Outcomes	
	Death	Clinical improvement
No. of events/no. of patients at risk (%)		
Vitamin C	12/70 (17.1)	52/70 (74.3)
No vitamin C	7/327 (2.1)	311/327 (95.1)
Crude analysis – hazard ratio (95% CI)	8.64 (3.40–21.94)	0.72 (0.53–0.96)
Propensity-score analyses – hazard ratio (95% CI)		
With inverse probability weighting^a^	2.69 (0.91–7.89)	0.76 (0.55–1.07)
With matching^b^	2.57 (0.39–16.79)	0.74 (0.48–1.14)

a The main analysis with a hazard ratio by a Cox regression model adjusted by basic stabilized inverse probability treatment weighting.

b The hazard ratio from a paired Cox regression.

### 3.3 Secondary outcome

**Figure 3:**
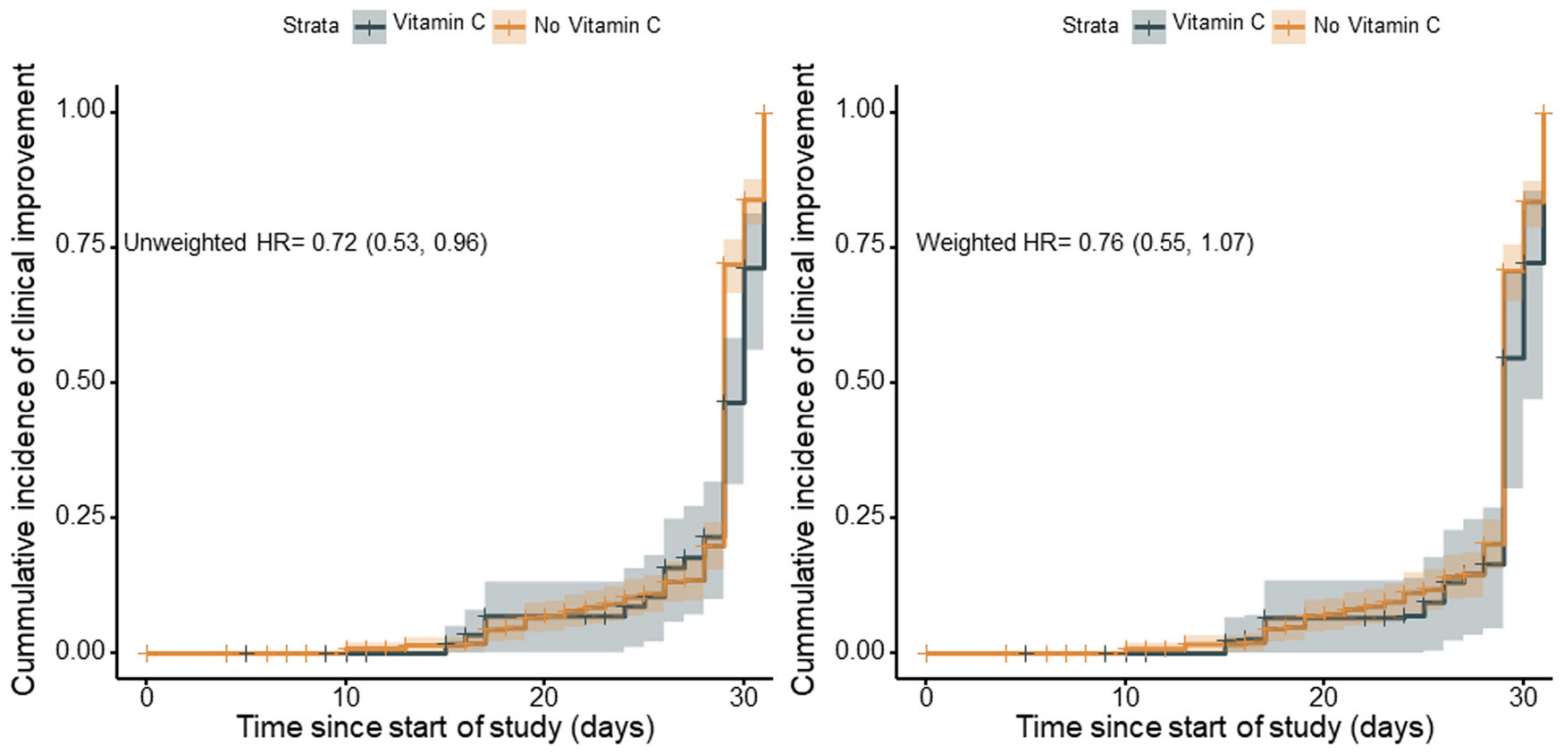
Kaplan–Meier curves for clinical improvement at the end of follow-up before and after inverse probability of treatment weighting (IPTW) analysis. On unadjusted analysis in the left panel, clinical improvement was less frequent among patients exposed to vitamin C than among those who were not exposed to it (HR, 0.72; 95% CI, 0.53–0.96). But no significant difference was observed between the two groups on IPTW analysis in the right panel (weighted HR, 0.76; 95% CI, 0.55–1.07). The shaded areas represent 95% CI.

By 15 March 2020, clinical outcomes of the 397 patients according to the 6-point ordinal scale are shown in Table A1. In the unadjusted analysis, the cumulative incidence of clinical improvement was 74.3% (52/70) among patients who received vitamin C, and 95.1% (311/327) among patients who did not receive it. Clinical improvement was less frequent among patients exposed to vitamin C than among those who were not exposed to it (HR, 0.72; 95% CI, 0.53–0.96) (Figure 3, left panel). However, there was no significant difference in the incidence of clinical improvement between two groups on IPTW analysis (weighted HR, 0.76; 95% CI, 0.55–1.07) (Figure 3, right panel) and on propensity-score matching analysis (HR, 0.74; 95% CI, 0.48–1.14) (Table 2).

### 3.4 Safety

During the follow-up period, adverse events which may be caused by vitamin C such as abdominal pain, diarrhea, or nausea [13] did not occur in patients with the use of vitamin C.

## 4 Discussion

In this study, based on the real-world data, we investigated the efficacy and safety of vitamin C use in a cohort of 397 patients with severe COVID-19 consecutively admitted to the hospital. We found that in addition to standard therapies, treatment with intravenous infusion of moderate doses of vitamin C (2–4 g/day) for at least 3 days, compared with standard therapies alone, did not result in significant differences in survival (weighted HR, 2.69; 95% CI, 0.91–7.89) or clinical improvement (weighted HR, 0.76; 95% CI, 0.55–1.07) during the follow-up period.

SARS-Cov-2 infection can cause a “cytokine storm” that can activate lung capillary endothelial cells followed by the infiltration of neutrophil and the enhancement of oxidative stress [1], which is exhibited by elevated levels of pro-inflammatory cytokines interleukin 1 (IL-1), IL-6, and tumor necrosis factor alpha, as well as the anti-inflammatory cytokine IL-10. The hyperinflammatory response is a major insult in pulmonary injury such as ARDS [14,15]. However, ARDS induced by cytokine storm may be the key cause of death in patients with COVID-19 [16]. A recent study found that the lungs of patients who died from COVID-19 owing to respiratory failure showed severe endothelial injury accompanied with the presence of intracellular virus [17].

Several biological effects of vitamin C in the critically ill management are reviewed recently [18]. For example, served as a radical oxygen scavenger, vitamin C could protect cells from oxidative stress. Vitamin C could also enhance neutrophil phagocytosis, affect macrophage migration, and may increase antibody formation. Moreover, vitamin C could decrease endothelium intercellular cell adhesion molecule expression and leukocyte adhesion, and improve endothelial cell function. It has been reported that vitamin C can reduce acute inflammatory lung injury induced by oxidative stress in patients receiving mechanical ventilation [19]. Previously, several studies concluded that vitamin C can shorten the length of stay in the ICU and the duration of mechanical ventilation in critically ill patients [7,8]. However, a recent randomized controlled trial (RCT) study on patients with sepsis and ARDS demonstrated that high-dose vitamin C (50 mg/kg four times a day for 4 days) use did not significantly ameliorate organ failure scores or alter the levels of biomarkers for inflammation compared with patients in placebo groups [20].

Recently, a single-center observational study including 17 patients who received intravenous vitamin C for COVID-19 showed that there was a significant decrease in inflammatory markers and a trend to decreasing FiO_2_ requirements after vitamin C use at a dose of 3 g/day for 3 days. However, the changes in risk of death or clinical improvement after vitamin C administration were not evaluated [21]. In the present study, we adjusted for several likely confounders, such as patients’ demographics, comorbidities, laboratory findings, and medications at study baseline. According to the Cox regression analysis with IPTW based on the propensity score, there was no significant association between treatment with moderate doses of intravenous vitamin C and risk of death or clinical improvement in patients with severe COVID-19. Propensity-score matching analysis yielded similar results. The possible causes that contributed to no beneficial role of vitamin C use in the treatment of severe COVID-19 were as follows.

The dose of vitamin C use is relatively small in our cohort. A previous study showed that in children a dose of vitamin C (1–2 g/day) reduced the length of the common cold by 18% [22]. The adult may need higher dose of vitamin C intake to effectively counter COVID-19. High-dose intravenous vitamin C (1.5 g/kg) has been recommended by the National Institute of Health experts as safe and without serious side effects for people with cancer [23]. Currently, the clinical efficacy of high-dose vitamin C used in severe COVID-19 is being investigated by multiple clinical trials [24]. Our study had been performed in a designated clinic center for COVID-19 during the outbreak in Wuhan, China. All patients with COVID-19 in this clinic center were transferred from other hospitals. The timing of vitamin C treatment initiation may not be appropriate because each patient with COVID-19 was at different stage of the disease on admission. Additionally, more than half of patients started vitamin C treatment 48 h after admission and the duration of vitamin C use for each patient was diversified. All of these would bias the results.

There are several limitations in this study. First, this is a single-center, small sample size, retrospective study, which limited to some extent the generalization of the results. Large-scale RCTs are needed to assess the effect of high-dose vitamin C in the treatment of COVID-19. Second, though we applied robust statistical methods to adjust for likely confounders in our analysis, the potential unmeasured confounders may still bias our results.

In this preliminary study, besides standard therapies, treatment with moderate doses of vitamin C intravenously for at least 3 days compared with standard therapies alone did not significantly improve the survival or clinical improvement among patients with severe COVID-19. RCTs of high-dose vitamin C use are warranted to further investigate its potential efficacy in the treatment of severe COVID-19.

## Appendix

**Table A1:** Clinical outcomes according to the 6-point ordinal scale

Characteristic	Vitamin C group	No vitamin C group
	(*n* = 70)	(*n* = 327)
Clinical status at study baseline – no. of patients (%)		
2: Breathing ambient air without supplemental oxygen	9 (12.9)	51 (15.6)
3: Receiving low-flow supplemental oxygen	53 (75.7)	273 (83.5)
4: Requiring noninvasive mechanical ventilation or high-flow oxygen	5 (7.1)	2 (0.6)
5: Requiring invasive ventilation or ECMO	3 (4.3)	1 (0.3)
Clinical status at the end of follow-up – no. of patients (%)		
1: Discharge	50 (71.4)	310 (94.8)
2: Hospitalized, breathing ambient air without supplemental oxygen	0	0
3: Hospitalized, receiving low-flow supplemental oxygen	2 (2.9)	7 (2.1)
4: Hospitalized, requiring noninvasive mechanical ventilation or high-flow oxygen	1 (1.4)	1 (0.3)
5: Hospitalized, requiring invasive ventilation or ECMO	5 (7.1)	2 (0.6)
6: Death	12 (17.2)	7 (2.2)
Clinical improvement at the end of follow-up – no. of patients (%)^a^	52 (74.3)	311 (95.1)

Abbreviations: ECMO = extracorporeal membrane oxygenation.

a Clinical improvement was defined as live discharge from the hospital, or a reduction of at least 2 points from baseline on a 6-point ordinal scale.
